# A Translational Study of a New Therapeutic Approach for Acute Myocardial Infarction: Nanoparticle-Mediated Delivery of Pitavastatin into Reperfused Myocardium Reduces Ischemia-Reperfusion Injury in a Preclinical Porcine Model

**DOI:** 10.1371/journal.pone.0162425

**Published:** 2016-09-07

**Authors:** Kenzo Ichimura, Tetsuya Matoba, Kaku Nakano, Masaki Tokutome, Katsuya Honda, Jun-ichiro Koga, Kensuke Egashira

**Affiliations:** 1 Department of Cardiovascular Medicine, Kyushu University Graduate School of Medical Sciences, Fukuoka, Japan; 2 Department of Cardiovascular Research, Development, and Translational Medicine, Center for Cardiovascular Disruptive Innovation, Kyushu University, Fukuoka, Japan; Rutgers New Jersey Medical School, UNITED STATES

## Abstract

**Background:**

There is an unmet need to develop an innovative cardioprotective modality for acute myocardial infarction, for which interventional reperfusion therapy is hampered by ischemia-reperfusion (IR) injury. We recently reported that bioabsorbable poly(lactic acid/glycolic acid) (PLGA) nanoparticle-mediated treatment with pitavastatin (pitavastatin-NP) exerts a cardioprotective effect in a rat IR injury model by activating the PI3K-Akt pathway and inhibiting inflammation. To obtain preclinical proof-of-concept evidence, in this study, we examined the effect of pitavastatin-NP on myocardial IR injury in conscious and anesthetized pig models.

**Methods and Results:**

Eighty-four Bama mini-pigs were surgically implanted with a pneumatic cuff occluder at the left circumflex coronary artery (LCx) and telemetry transmitters to continuously monitor electrocardiogram as well as to monitor arterial blood pressure and heart rate. The LCx was occluded for 60 minutes, followed by 24 hours of reperfusion under conscious conditions. Intravenous administration of pitavastatin-NP containing ≥ 8 mg/body of pitavastatin 5 minutes before reperfusion significantly reduced infarct size; by contrast, pitavastatin alone (8 mg/body) showed no therapeutic effects. Pitavastatin-NP produced anti-apoptotic effects on cultured cardiomyocytes in vitro. Cardiac magnetic resonance imaging performed 4 weeks after IR injury revealed that pitavastatin-NP reduced the extent of left ventricle remodeling. Importantly, pitavastatin-NP exerted no significant effects on blood pressure, heart rate, or serum biochemistry. Exploratory examinations in anesthetized pigs showed pharmacokinetic analysis and the effects of pitavastatin-NP on no-reflow phenomenon.

**Conclusions:**

NP-mediated delivery of pitavastatin to IR-injured myocardium exerts cardioprotective effects on IR injury without apparent adverse side effects in a preclinical conscious pig model. Thus, pitavastatin-NP represents a novel therapeutic modality for IR injury in acute myocardial infarction.

## Introduction

Coronary heart disease is the leading cause of death worldwide, and acute myocardial infarction (AMI) is the most severe manifestation of this disease[1]. Myocardial infarct (MI) size is a major determinant of the clinical outcomes and prognosis in patients with AMI[2], and early reperfusion therapy is a standard strategy to reduce MI size. However, reperfusion induces ischemia-reperfusion (IR) injury, which reduces the therapeutic effects of reperfusion therapy[3]. Therefore, there is an unmet need to develop new cardioprotective modalities to reduce IR injury.

In previous studies, we exploited the cardioprotective effects of the 3-hydroxy-3-methylglutaryl coenzyme-A reductase inhibitors (statins)[4] to engineer bioabsorbable poly(lactic acid/glycolic acid) (PLGA) polymers loaded with pitavastatin (pitavastatin-NP)[5–14] and showed that intravenous treatment with pitavastatin-NP at the time of reperfusion exerts a cardioprotective effect in rats subjected to IR injury[9]. This cardioprotective effect was associated with activation of the PI3K-Akt pathway and reduced inflammation[9]. Although our results in a rat model showed the efficacy of pitavastatin-NP in IR injury reduction, an assessment of the efficacy and safety of pitavastatin-NP in a large animal model, particularly with regard to its safety in hemodynamics and coronary circulation, is necessary to translate our previous findings to clinical medicine.

Recently, porcine models have gained recognition as an effective preclinical large animal IR model to examine the effects of various drugs and interventions on IR injury and the resulting MI size[15,16]. However, there are some methodological problems associated with large animal IR models. First, most studies have been performed under general anesthesia, which may affect sympathetic nerve activity, hemodynamic status, cardiac function, and, subsequently, MI size. Moreover, while some anesthetics exert cardiotoxic side effects, which can exacerbate IR injury[16,17], others exert cardioprotective effects on IR injury[18,19]. Second, anesthetized porcine models of myocardial ischemia display high mortality rates due to fatal arrhythmia, such as ventricular fibrillation, within 24 hours of ischemia[20–23], which may introduce bias into results based on the exclusion of dead animals.

In the present study, to overcome these problems, we developed a novel conscious mini-pig myocardial IR injury model and performed a preclinical proof-of-concept study to test the hypothesis that pitavastatin-NP is a safe and effective therapeutic modality that can offer cardioprotection against IR injury. We used Bama mini-pigs because the metabolism of statins in this animal are similar to those in humans[24]. In addition, we performed exploratory analyses such as pharmacokinetics and the effects on no-reflow phenomenon in anesthetized pig model.

## Materials and Methods

### Preparation of PLGA Nanoparticles

PLGA with an average molecular weight of 20,000 Da and a copolymer ratio of lactide to glycolide of 75:25 (Wako Pure Chemical Industries, Osaka, Japan) was used as a matrix for the nanoparticles, and polyvinyl alcohol (PVA-403; Kuraray, Osaka, Japan) was used as a dispersing agent. PLGA nanoparticles incorporating the fluorescent marker fluorescein-isothiocyanate (FITC; Dojin Chemical, Tokyo, Japan) (FITC-NP) or pitavastatin (Kowa Pharmaceutical, Tokyo, Japan) (pitavastatin-NP) were prepared by the emulsion solvent diffusion method in purified water, as previously described[9,25]. The FITC-NP and pitavastatin-NP contained 4.2% (wt/vol) FITC and 12.0% (wt/vol) pitavastatin, respectively. A sample of the NP suspension in distilled water was used for particle size analysis. The average diameters of FITC-NP and pitavastatin-NP were 231 nm and 159 nm, respectively. Surface charge (zeta potential) was also analyzed using a Zetasizer Nano system (Sysmex, Hyogo, Japan) revealing an anionic nature [-16.7 mV (FITC-NP) and -4.1 mV (pitavastatin-NP)].

### Care and Use of Animals

We utilized two types of animals in the present study; Bama mini-pigs and domestic pigs. The experiment using Bama mini-pig was conducted in JOINN laboratories Inc. (http://www.joinnlaboratories.com/home/index2.html), in Suzhou, China. JOINN was accredited by AAALAC in 2011 by China FDA. This experiment was reviewed and approved by the local ethics committee in JOINN laboratories. The experiments using domestic pigs were conducted in Kyushu University, reviewed and approved by the Committee of Ethics of Animal Experiments at Kyushu University Graduate School of Medical Science. Both experiments were conducted according to the guidelines of the Guide for the Care and Use of Laboratory Animals from the National Institutes of Health (NIH).

Animals were kept in a separate cage in a room which temperature was kept between 18 to 26°C, humidity between 40 to 70% and the lights were kept on between 6:30 am to 6:30 pm. Normal chow diet were given twice daily and water was supplied ad libitum. The physical condition of animals was checked twice daily, and 6 times daily after surgical preparation. Veterinary physicians with expertise to perform the pig experiments were asked to attend and observe the course of experiment. At the end of each experiments, animals were euthanized by an overdose of pentobarbital.

### Conscious Mini-Pig Model of IR injury

Eighty-four Bama mini-pigs[24] (aged 4–6 months, weight 10–15 kg) were used in this study. The animals were anesthetized with ketamine hydrochloride (20 mg/kg i.m.; Daiichi-Sankyo Propharma, Tokyo, Japan) and xylazine (3.5 mg/kg, Sigma-Aldrich, MO) and were maintained under anesthesia with isoflurane (1% to 2.5%, Abbvie, IL) using a ventilator after intubation. Under aseptic conditions, a left thoracotomy was performed, and a pneumatic cuff occluder (VO-2, Docxs Biomedical Products and Accessories, CA) was placed at the proximal portion of the left circumflex coronary artery (LCx). A telemetry transmitter system (TL11M2-D70-PCT, Data Sciences International Inc., MN) was implanted in the subcutaneous space of the abdomen, and the tip of the manometer was placed in the abdominal aorta through the left femoral artery. After the chest was closed, the animals were allowed to recover from surgery. On the day after surgery, the LCx was occluded for 60 minutes by inflating the cuff, and then the tissue was reperfused by deflating the cuff. Transmural ischemia was confirmed by observing ST segment elevation by electrocardiography. Five minutes prior to reperfusion, the pigs were divided into four groups treated with intravenous injection of one of the following drugs: 1) saline (10 mL/body), 2) FITC-NP (PLGA containing 10 mg of FITC in saline; 10 mL/body), 3) pitavastatin alone (8 mg in saline; 10 mL/body), or 4) pitavastatin-NP (PLGA containing 4, 8, 16, or 32 mg of pitavastatin in saline; 10 mL/body). Before and during the IR procedure, an electrocardiogram at the left precordial lead, arterial blood pressure, heart rate and body temperature were continuously monitored using a telemetry system. Blood samples were collected at baseline (1 hour prior to ischemia) and 24 hours after reperfusion.

To measure MI size, the animals were euthanized 24 hours after reperfusion with an overdose of pentobarbital, and the heart was excised (S1A Fig). The LCx was re-occluded, and the MI size was measured using 1.0% Evans blue (Sigma-Aldrich) and 1.0% triphenyltetrazolium chloride staining (TTC, Sigma-Aldrich), as previously described[23]. For western blot and immunohistochemical analyses, pigs treated with FITC-NP (PLGA containing 10 mg of FITC in saline; 10 mL/body) or pitavastatin-NP (PLGA containing 16 mg of pitavastatin in saline; 10 mL/body) were euthanized after 2 or 24 hours of reperfusion, and the tissues were harvested from the non-ischemic and ischemic areas (S1B and S1C Fig). Seven pigs each from the saline (10 mL/body) or pitavastatin-NP (PLGA containing 16 mg of pitavastatin in saline; 10 mL/body)-treated groups were monitored until 4 weeks after reperfusion, and then cardiac MRI was performed to assess cardiac function and left ventricular remodeling (S1D Fig).

#### Assessment of Cardiac Function and remodeling by Magnetic Resonance Imaging (MRI)

Cardiac function and remodeling were assessed using a cardiac 3.0 Tesla MRI (MAGNETOM, Siemens Medical Solutions, Erlangen, Germany) 4 weeks after IR injury in the saline- or pitavastatin-NP-treated (16 mg/body) animals (S1D Fig). The animals were injected with a bolus (equal volumes at a concentration of 0.075 mmol/kg) of a 1 M gadolinium-based contrast agent (gadobutrol/Gadovist, Berlin-Wedding, Schering, Germany) at 4 mL/s, followed by a 20-mL saline flush using a power injector via another venous line. The left ventricular ejection fraction (LVEF) was manually calculated by tracing the endocardial borders at end systole and end diastole for each short axis slice. The LV mass was calculated according to the equation LV mass = Σ(slice thickness×muscle size×myocardial density), as previously described[26].

#### Western Blot Analysis

Tissues were harvested from the non-ischemic and ischemic areas and were snap frozen in liquid nitrogen. The samples were homogenized using a cryopress (CP-100W, Microtec, Chiba, Japan) in lysis buffer containing 10 mmol/L Tris-HCl, pH 7.4, 5 mmol/L EDTA, 50 mmol/L NaCl, 30 mmol/L sodium phosphate, 50 mmol/L NaF, 1% Triton X-100, a protease inhibitor cocktail (Thermo Fisher Scientific, MA) and a phosphatase inhibitor cocktail (Thermo Fisher Scientific, MA). Tissue lysates (10 μg) were separated on 7.5% polyacrylamide gels and blotted onto polyvinylidene fluoride membranes (Merck Millipore, MA). Protein expression was analyzed using antibodies against Akt (Cell Signaling Technology, MA), p-Akt (Cell Signaling Technology, MA) or GAPDH (Santa Cruz Biotechnology, CA). Immune complexes were visualized with horseradish peroxidase-conjugated secondary antibodies. The bound antibodies were detected by chemiluminescence with the ECL Prime western blotting detection system (GE Healthcare, CT) and were quantified by densitometry.

#### Immunohistochemistry

Tissues from the non-ischemic and ischemic areas were processed by routine paraffin embedding. The degree of oxidative stress was assessed by immunohistochemical staining of 5-μm sections with anti-8-hydrooxy-2’deoxyguanosine (8-OHdG) (dilution 1:40, Nikken Seil, Shizuoka, Japan) and anti-4-hydroxy-2-nonenal (4-HNE) (dilution 1:20, Nikken Seil). Apoptotic nuclei were detected by terminal deoxynucleotidyl-transferase mediated dUTP nick-end labeling (TUNEL) staining using the In Situ Apoptosis Detection Kit (Takara Bio, Shiga, Japan) according to the instructions recommended by the manufacturer.

### Anesthetized Domestic Pig Model of IR Injury

Domestic male pigs (Kyudo, Tosu, Japan; aged 4–6 months, weight 25–35 kg) were anesthetized with ketamine hydrochloride (15 mg/kg, i.m.) and xylazine (3.5 mg/kg) and were maintained under anesthesia with isoflurane (5.0%) administered through a facemask. The pigs were then intubated and mechanically ventilated with ambient air and isoflurane (1.0–2.0%). A mid-sternal thoracotomy was performed, and the LCx was occluded for 1 hour, followed by reperfusion. Seven pigs were treated with saline, FITC (0.33 mg/kg in saline; 10 mL/body), or FITC-NP (PLGA containing 0.33 mg/kg FITC in saline; 10 mL/body) 5 minutes before reperfusion, and 2 hours after reperfusion, the pigs were euthanized, and the hearts were excised. The LCx was re-occluded, and 1.0% Evans blue was injected into both coronary arteries. The left ventricles were sectioned into 10-mm-thick cross-sectional myocardial slices and were photographed by fluorescence stereomicroscopy (SteREO Lumar V12, Zeiss, Oberkochen, Germany). Tissues from the non-ischemic or ischemic areas were evaluated by fluorescence microscopy (BX53, Olympus, Tokyo, Japan) (S2A Fig).

The other 10 pigs were intravenously injected with pitavastatin (16 mg/body in saline; 10 mL/body) or pitavastatin-NP (PLGA containing 16 mg of pitavastatin in saline; 10 mL/body) 5 minutes prior to reperfusion. Serial blood sampling and biopsy of ischemic and non-ischemic myocardium was performed at 5, 15, 30 or 60 minutes after reperfusion to measure the plasma and tissue concentrations of pitavastatin (S2B Fig). Biopsy specimens from the heart was harvested by using a 1.5 mm biopsy punch (BP-15F, Kai Industries Co., Ltd, Tokyo, Japan).

#### Measurement of Pitavastatin Concentrations in Plasma and Myocardial Tissue

The pitavastatin concentrations in the plasma and heart were measured by liquid chromatography coupled to tandem mass spectrometry (LC/MS/MS), as previously described[9]. Briefly, the high-performance liquid chromatography (HPLC) analysis was performed using an Agilent 1100 series system (Agilent Technologies, Inc., CA). The column temperature was maintained at 40°C. The flow rate was 0.3 mL/min. Pre-prepared plasma or tissue homogenate sample solutions were injected from the autosampler into the HPLC system. The turbo ion spray interface was operated in the positive ion mode at 4,800 V and 550°C. The analytical data were processed using Analyst software (version 1.4m, Applied Biosystems, CA).

#### Evaluation of the No-reflow Phenomenon

Another group of domestic male pigs that had been treated with saline (10 mL/body) or pitavastatin-NP (PLGA containing 16 mg of pitavastatin in saline; 10 mL/body) were reperfused for 2 hours (S2C Fig), and 4% thioflavin S (Sigma-Aldrich) was injected into the left coronary artery via a 7-Fr catheter[27]. Then, the pigs were euthanized, and the hearts were excised. The LCx was re-occluded, and 1% Evans blue was injected into both coronary arteries. The left ventricles were sectioned into 10-mm-thick cross-sectional myocardial slices and were photographed using a digital camera (EOS Kiss XIIa, Canon, Tokyo, Japan) and an Image Quant LAS 4010 (GE Healthcare, CT) using 365 nm UV epi-illumination and an L41 UV filter.

### Preparation of Cultured Cardiomyocytes, Confocal Imaging and Flow Cytometric Analysis

A primary culture of neonatal rat ventricular myocytes was prepared from the ventricles of neonatal Sprague-Dawley rats (Kyudo), as previously described[28]. Briefly, neonatal rats were euthanized under anesthesia with isoflurane, after which the hearts were rapidly excised and digested with 0.05% trypsin (Thermo Fischer Scientific) and collagenase type 2 (Worthington, NJ). After digestion, the cells were suspended in Dulbecco’s Modified Eagle Medium (DMEM, Thermo Fischer Scientific) containing 10% fetal bovine serum (FBS, Thermo Fischer Scientific) and 1% penicillin/streptomycin (Thermo Fischer Scientific) and were plated twice in 100-mm culture dishes (Cellstar, Greiner Bio-One, NY) for 70 minutes each to reduce the number of non-myocytes. The non-adherent cells were then plated in 35-mm culture dishes (Primaria, BD Bioscience, CA) at a density of 1.0×10^6^ cells/3 mL DMEM. Myocytes were maintained at 37°C in humidified air with 5% CO_2_ for 36 hours after plating on culture dishes.

Cultured myocytes were incubated in DMEM without glucose (Sigma-Aldrich) in a hypoxic chamber (1% O_2_) for 12 hours. The following experimental protocols were performed after the hypoxic period.

Experimental protocol 1: The culture medium was exchanged with normal DMEM containing the following drugs: 1) pitavastatin alone (0.1, 1.0, or 10 μmol/L), 2) pitavastatin-NP (0.1, 1.0, or 10 μmol/L), and 3) pitavastatin-NP (0.1, 1.0, or 10 μmol/L) with mevalonic acid (100 μmol/L, Sigma-Aldrich). The myocytes were further cultured in humidified air with 5% CO_2_ for 12 hours. After the incubation period, the myocytes were washed with cold Dulbecco’s phosphate-buffered saline (DPBS, Thermo Fisher Scientific) twice, dissociated from the dish using 1 mL of accutase (Innovative Cell Technologies, CA), and cell death was assessed by flow cytometry (Galios, Beckman Coulter, CA) using the FITC annexin-V apoptosis detection kit (BD Biosciences) according to the instructions recommended by the manufacturer, as previously described[29].

Experimental protocol 2: The culture medium was exchanged with normal DMEM containing FITC (10 μmol/L) or FITC-NP (10 μmol/L). The myocytes were further cultured in humidified air with 5% CO_2_ for 12 hours. After the incubation period, the myocytes were washed with cold DPBS twice, fixed with methanol at -20°C for 20 min, and mounted with medium containing DAPI (Vectashield, Vector Laboratories, CA). Images of the cardiomyocytes were captured by confocal microscopy (FV1000, OLYMPUS, Tokyo, Japan).

### Statistical Analysis

The data are expressed as either means ± SD or means ± SEM. The statistical analysis of differences between two groups was assessed using an unpaired *t*-test, and differences between three or more groups were assessed using ANOVA and multiple comparison tests featured in Prism Software version 6.0 (Graph Pad Software, CA). P-values <0.05 were considered statistically significant.

## Results

### Cardioprotective Effects of Pitavastatin-NP in Conscious Mini-Pigs

No animals died during the surgical preparations before the IR experiments. One animal died during the induction of ischemia (S3A Fig).

**Fig 1:**
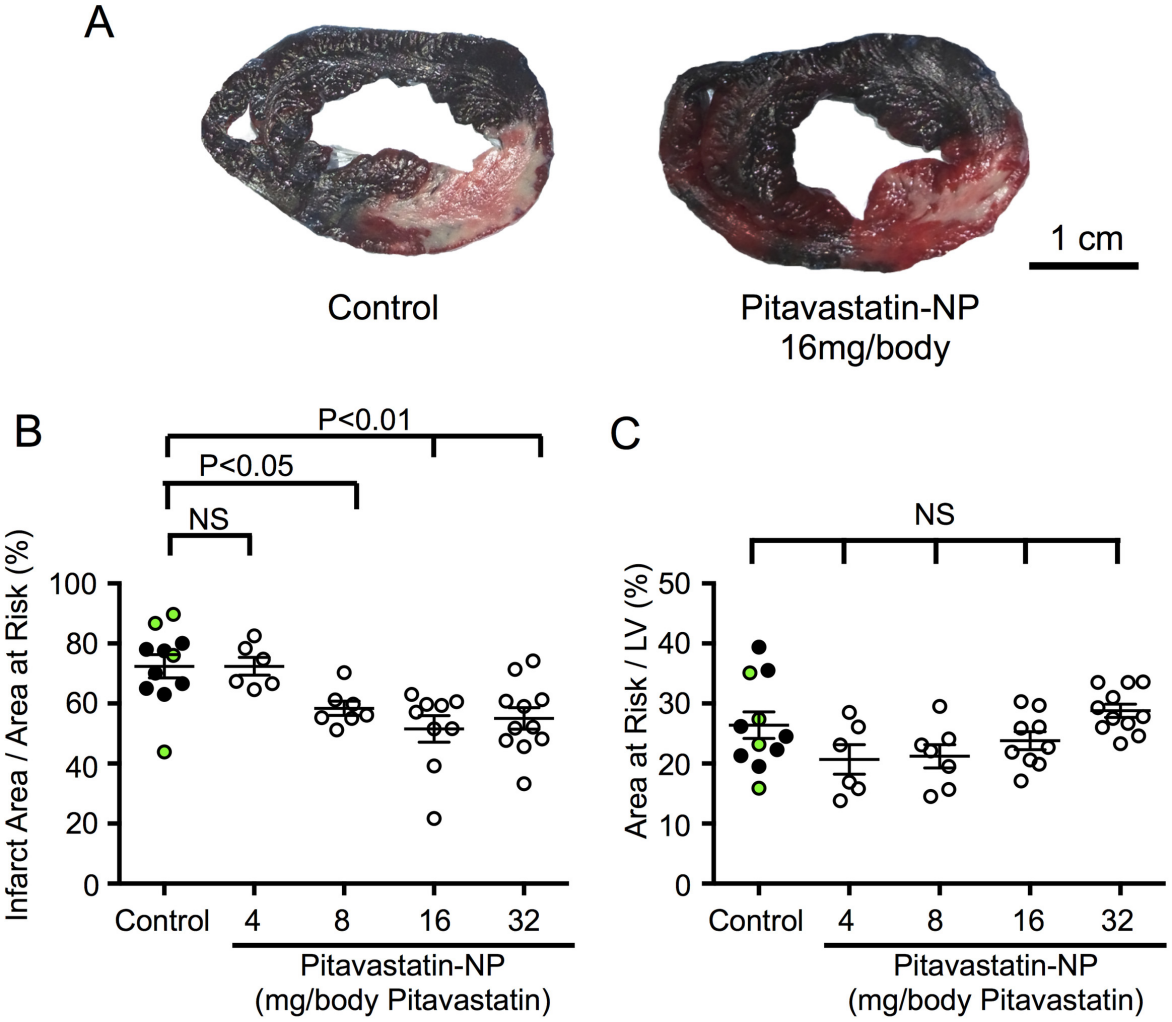
The Effect of Pitavastatin-NP on MI Size in Mini-Pigs. **(A)** Representative photographs of heart cross-sections 24 hours after reperfusion in control (saline- or FITC-NP-treated) groups and pitavastatin-NP-treated groups. The blue areas are stained by Evans blue and indicate non-ischemic areas, whereas the red areas are stained by TTC and indicate areas at risk without infarction. The pale areas indicate the infarcted areas. Scale bar: 1 cm. **(B)** The effects of pitavastatin-NP on MI size. The black dots in the control group show the data of saline-treated animals, whereas green dots are those of FITC-NP-treated animals. The data are expressed as means ± SEM (n = 6–11 each) and were compared using one-way ANOVA followed by Dunnett’s multiple comparison test. **(C)** The area at risk as a percentage of the left ventricle (LV). The data are expressed as means ± SEM (n = 6–11 each) and were compared using one-way ANOVA followed by Dunnett’s multiple comparison test.

We observed no difference in mortality during the 24 hours of reperfusion among the saline- (n = 7), FITC-NP- (n = 4), pitavastatin- (n = 3), and pitavastatin-NP-treated (n = 33) groups (S3A Fig). Compared with the control group (saline and FITC-NP groups), intravenous treatment with pitavastatin-NP containing 8, 16 or 32 mg/kg pitavastatin at the time of reperfusion significantly reduced the MI size (Fig 1A and 1B). In contrast, treatment with pitavastatin-NP containing 4 mg/kg of pitavastatin did not reduce the MI size (Fig 1B). The percentage of area at risk in the left ventricle was comparable between all study groups (Fig 1C). There was no significant difference in the MI size between the saline-treated and FITC-NP-treated animals (S4A and S4B Fig). Treatment with pitavastatin alone at a dose of 8 mg/body did not reduce the MI size compared with the same dose of pitavastatin-NP (S4C and S4D Fig).

**Fig 2:**
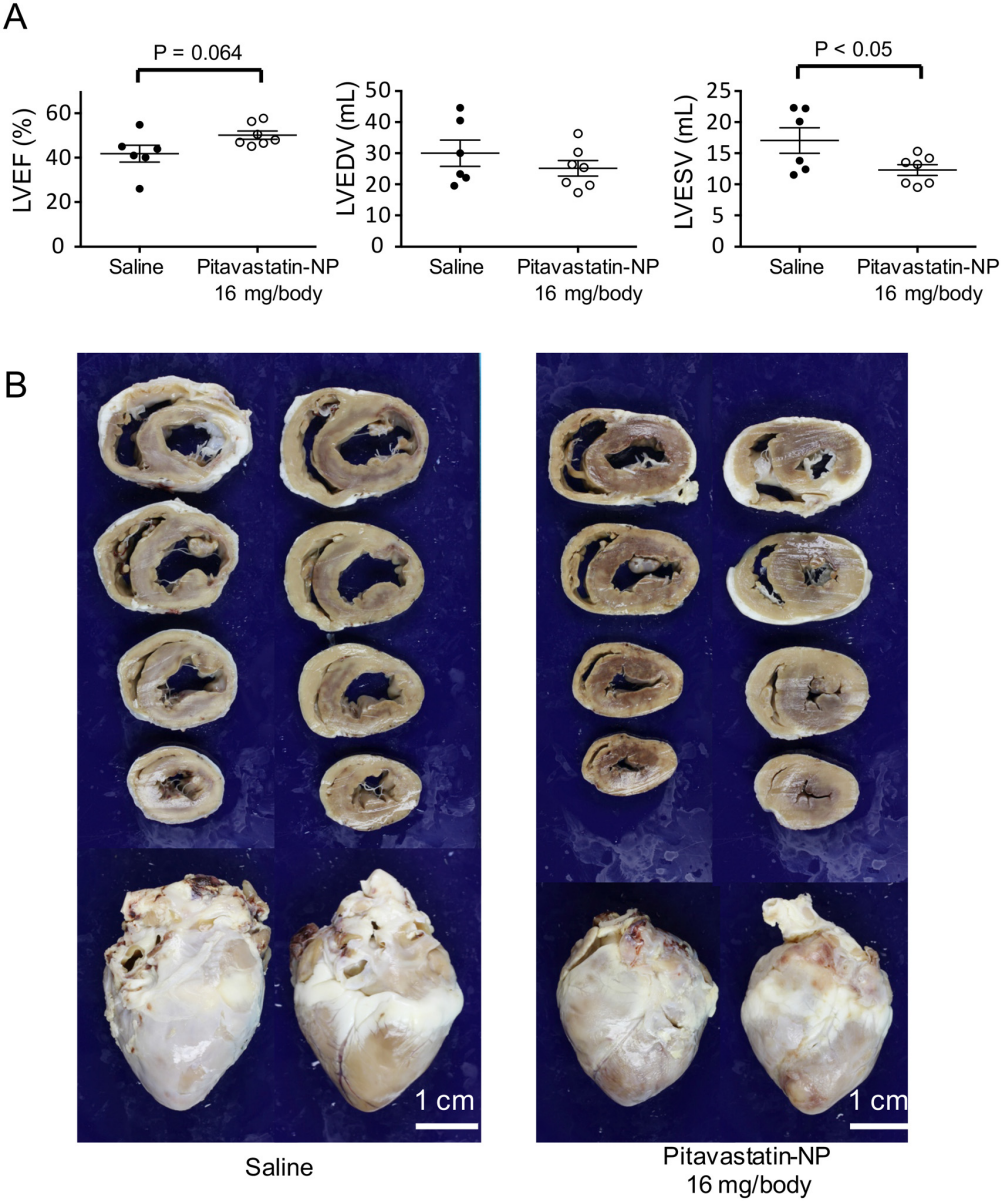
Assessment of Left Ventricular Ejection Fraction (LVEF) and Left Ventricular Remodeling by MRI in Mini-Pigs. **(A)** LVEF, left ventricular end diastolic volume (LVEDV) and left ventricular end systolic volume (LVESV) 4 weeks after reperfusion in the saline- or pitavastatin-NP (16 mg/body pitavastatin)-treated groups were measured by cardiac MRI. The data are expressed as means ± SEM (n = 6–7 each) and were compared using an unpaired *t*-test. **(B)** Representative photographs of heart cross-sections 4 weeks after reperfusion in the saline- and pitavastatin-NP-treated groups. Scale bar: 1 cm.

Cardiac MRI performed 4 weeks after IR injury showed that treatment with pitavastatin-NP (16 mg/body of pitavastatin) tended towards an improvement in the left ventricular ejection fraction, although these results did not reach statistical significance: 50.1 ± 5.0% in the pitavastatin-NP group and 41.8 ± 9.3% in the saline group (P = 0.064, n = 6–7 each) (Table 1, Fig 2A). The left ventricular end systolic volume was significantly decreased in the pitavastatin-NP-treated group, 12.3 ± 2.3 mL in the pitavastatin-NP group and 17.1 ± 5.0 mL in the saline group (P<0.05, n = 6–7 each), which suggests that the remodeling of the left ventricle was improved by pitavastatin-NP treatment (Fig 2A and 2B).

**Table 1:** Cardiac MRI studies.

	Saline	Pitavastatin-NP 16 mg/body
LVEDV (mL)	30.0 ± 4.2	25.1 ± 2.5
LVESV (mL)	17.1 ± 2.1	12.3 ± 0.9 *
LVEF (%)	41.8 ± 3.8	50.1 ± 1.9
LVEDV index (mL/kg)	1.66 ± 0.18	1.66 ± 0.11
LVESV index (mL/kg)	0.96 ± 0.11	0.82 ± 0.05
LV mass (g)	43.2 ± 3.7	36.5 ± 3.1
LV mass index (g/kg)	2.43 ± 0.20	2.42 ± 0.12

The data are expressed as the mean ± SD (n = 6–7 each).

LVEDV: left ventricular end diastolic volume, LVESV: left ventricular end systolic volume, LVEF: left ventricular ejection fraction, LV: left ventricular

* P<0.05 versus control using t-test.

We observed no significant differences in blood pressure and heart rate among the saline-, FITC-NP-, pitavastatin- and pitavastatin-NP-treated groups (Table 2). Moreover, we observed no significant difference in the incidence of ventricular arrhythmias (premature ventricular contraction and non-fatal ventricular tachycardia) between the treatment groups (Table 3). We observed no significant difference between all treated groups with regard to liver function, renal function and lipid metabolism (Table 4).

**Table 2:** Blood pressure and heart rate during ischemia-reperfusion in conscious mini-pig model.

**Systolic Blood Pressure (mmHg)**									
Groups	Dose (/body)	Baseline	Ischemia	Reperfusion					
				0 min	1 hr	3 hr	5 hr	8 hr	24 hr
Saline	-	116 ± 9	105 ± 8	101 ± 7	100 ± 7	99 ± 12	98 ± 15	102 ± 11	98 ± 6
Pitavastatin	8 mg	115 ± 9	113 ± 9	119 ± 9	114 ± 20	123 ± 30	122 ± 23	123 ± 30	128 ± 36
FITC-NP	10 mg	107 ± 10	100 ± 3	103 ± 9	90 ± 20	85 ± 17*	91 ± 17	96 ± 15	96 ± 11
	4 mg	104 ± 16	113 ± 9	106 ± 7	112 ± 9	108 ± 12	110 ± 10	113 ± 12	120 ± 5
Pitavastatin-NP	8 mg	105 ± 12	106 ± 15	108 ± 13	109 ± 14	106 ± 16	108 ± 7	111 ± 16	118 ± 15
	16 mg	113 ± 28	107 ± 5	102 ± 7	97 ± 0	95 ± 7	99 ± 2	103 ± 10	108 ± 16
**Diastolic Blood Pressure (mmHg)**									
Groups	Dose (/body)	Baseline	Ischemia	Reperfusion					
				0 min	1 hr	3 hr	5 hr	8 hr	24 hr
Saline	-	70 ± 7	72 ± 10	66 ± 6	58 ± 7	61 ± 5	64 ± 8	69 ± 7	64 ± 11
Pitavastatin	8 mg	73 ± 11	76 ± 3	84 ± 5	78 ± 19	65 ± 16	81 ± 12	87 ± 18	79 ± 15
FITC-NP	10 mg	67 ± 2	67 ± 6	73 ± 17	46 ± 26	58 ± 11	62 ± 16	64 ± 11	60 ± 11
	4 mg	72 ± 16	74 ± 7	72 ± 15	71 ± 6	71 ± 10	74 ± 7	79 ± 18	82 ± 7
Pitavastatin-NP	8 mg	61 ± 15	68 ± 14	78 ± 21	68 ± 15	72 ± 24	70 ± 8	70 ± 16	84 ± 12
	16 mg	65 ± 34	63 ± 12	67 ± 4	64 ± 1	64 ± 7	67 ± 3	70 ± 4	78 ± 19
**Mean Blood Pressure (mmHg)**									
Groups	Dose (/body)	Baseline	Ischemia	Reperfusion					
				0 min	1 hr	3 hr	5 hr	8 hr	24 hr
Saline	-	86 ± 5	83 ± 9	78 ± 6	72 ± 4	74 ± 6	75 ± 10	80 ± 8	75 ± 8
Pitavastatin	8 mg	87 ± 10	88 ± 5	96 ± 1	90 ± 20	90 ± 11	95 ± 16	99 ± 21	95 ± 12
FITC-NP	10 mg	81 ± 4	78 ± 5	83 ± 14	61 ± 24	67 ± 13	72 ± 16	74 ± 712	72 ± 10
	4 mg	83 ± 16	87 ± 8	83 ± 12	85 ± 7	84 ± 11	86 ± 8	90 ± 16	94 ± 6
Pitavastatin-NP	8 mg	76 ± 14	81 ± 14	88 ± 18	82 ± 14	84 ± 21	83 ± 48	84 ± 16	96 ± 13
	16 mg	81 ± 32	78 ± 10	78 ± 5	75 ± 1	75 ± 7	77 ± 1	81 ± 6	88 ± 18
**Heart Rate (bpm)**									
Groups	Dose (/body)	Baseline	Ischemia	Reperfusion					
				0 min	1 hr	3 hr	5 hr	8 hr	24 hr
Saline	-	127 ± 8	130 ± 14	117 ± 13	110 ± 10	116 ± 15	116 ± 15	106 ± 5	114 ± 18
Pitavastatin	8 mg	125 ± 7	121 ± 5	119 ± 10	113 ± 10	121 ± 11	118 ± 9	136 ± 2	114 ± 15
FITC-NP	10 mg	123 ± 21	114 ± 9	119 ± 2	100 ± 26	106 ± 40	98 ± 33	104 ± 44	102 ± 18
	4 mg	118 ± 10	114 ± 4	106 ± 8	110 ± 13	110 ± 7	110 ± 13	128 ± 9	112 ± 7
Pitavastatin-NP	8 mg	139 ± 13	128 ± 32	131 ± 43	106 ± 14	108 ± 17	114 ± 11	110 ± 14	110 ± 12
	16 mg	120 ± 23	130 ± 9	111 ± 15	99 ± 11	106 ± 23	115 ± 21	117 ± 31	109 ± 4

The data are expressed as the mean ± SD (n = 3–6 each).

* P<0.05 versus pitavastatin (3 hr) using two-way ANOVA followed by Bonferroni’s multiple comparison test.

**Table 3:** Numbers of arrhythmias during ischemia-reperfusion in conscious mini-pig model.

Groups	Dose (/body)	Ischemia		Reperfusion					
		0 ~ 1 hr		0 ~ 1 hr		1 ~ 4 hr		4 ~ 24 hr	
		VT*	PVC**	VT*	PVC**	VT*	PVC**	VT*	PVC**
Saline	-	0.8 ± 0.6	39 ± 26	122 ± 62	121 ± 54	21 ± 8	45 ± 19	0 ± 0	6 ± 3
Pitavastatin	8 mg	1.3 ± 1.2	16 ± 14	81 ± 50	105 ± 65	33 ± 23	48 ± 32	0 ± 0	11 ± 8
FITC-NPs	10 mg	7 ± 7	46 ± 38	104 ± 55	96 ± 11	24 ± 14	46 ± 22	0 ± 0	10 ± 4
Pitavastatin-NPs	4 mg	10 ± 6	145 ± 106	42 ± 24	139 ± 74	8 ± 5	37 ± 21	0 ± 0	20 ± 15
	8 mg	0 ± 0	4.9 ± 2.3	137 ± 88	141 ± 43	20 ± 11	31 ± 6	0 ± 0	12 ± 5
	16 mg	1.2 ± 1.2	23 ± 20	102 ± 46	67 ± 24	10 ± 6	29 ± 12	0 ± 0	6 ± 4

The data are expressed as the mean ± SD (n = 3–6 each).

* VT: >3 consecutive ventricular beats.

** PVC: premature ventricular contraction

**Table 4:** Liver function, renal function and lipid profiles.

	Time (after Reperfusion)	Saline	Pitavastatin 8 mg/body	Pitavastatin-NP (/body)			
				4 mg	8 mg	16 mg	32 mg
AST (IU/L)	- 2hr	72 ± 2	57 ± 11	97 ± 42	99 ± 18	105 ± 13	72 ± 10
	24 hr	56 ± 2	32 ± 2	41 ± 10	72 ± 14	79 ± 17	51 ± 4
ALT (IU/L)	- 2hr	67 ± 4	62 ± 1	60 ± 11	79 ± 9	53 ± 5	54 ± 4
	24 hr	76 ± 5	53 ± 6	57 ± 8	90 ± 14	60 ± 5	60 ± 2
BUN (mg/dL)	- 2hr	10 ± 1	16 ± 1	14 ± 3	12 ± 1	15 ± 2	10 ± 1
	24 hr	9 ± 1	7 ± 0	8 ± 2	9 ± 1	11 ± 1	7 ± 1
Creatinin (mg/dL)	- 2hr	0.5 ± 0.0	0.6 ± 0.0	0.5 ± 0.1	0.5 ± 0.1	0.6 ± 0.0	0.4 ± 0.0
	24 hr	0.5 ± 0.0	0.5 ± 0.1	0.5 ± 0.0	0.5 ± 0.0	0.5 ± 0.0	0.4 ± 0.0
Total cholesterol (mg/dL)	- 2hr	54 ± 5	50 ± 3	43 ± 6	45 ± 10	59 ± 4	42 ± 3
	24 hr	75 ± 4	72 ± 5	70 ± 6	76 ± 5	74 ± 8	66 ± 4
Triglyceride (mg/dL)	- 2hr	15 ± 3	20 ± 3	11 ± 3	10 ± 1	16 ± 4	12 ± 3
	24 hr	36 ± 16	19 ± 1	17 ± 1	19 ± 2	28 ± 8	30 ± 5

The data are expressed as the mean ± SEM (n = 3–6 each).

**Fig 3:**
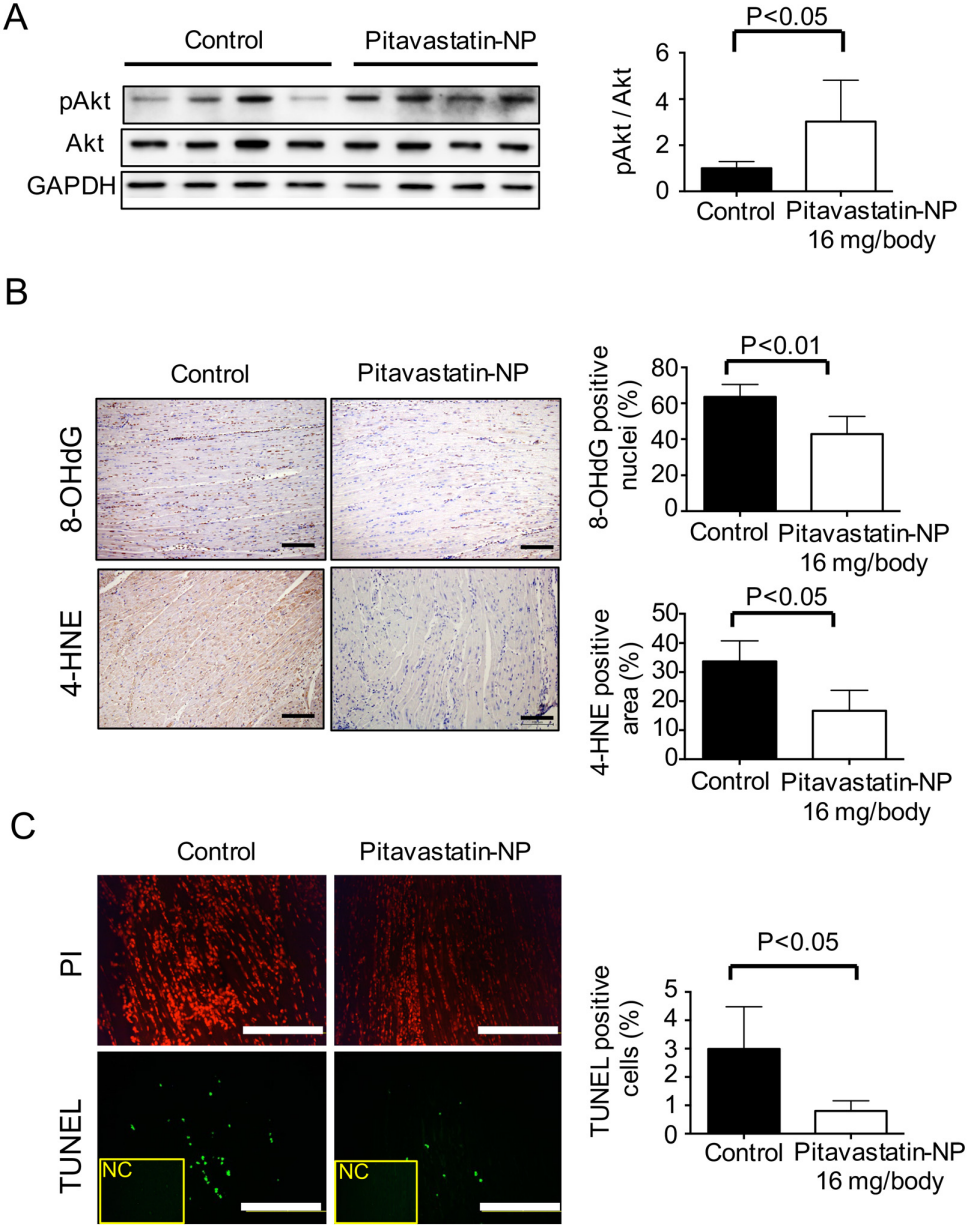
The Effect of Pitavastatin-NP on Akt Phosphorylation, Oxidative Stress and Apoptosis in Mini-Pigs. **(A)** Tissues from the ischemic and non-ischemic areas were analyzed by immunoblotting for phosphorylated Akt in the control and pitavastatin-NP (16 mg/body of pitavastatin) groups. The bar graph indicates the ratio of phosphorylated Akt to Akt. The data are expressed as means ± SD (n = 4–5 each) and were compared using an unpaired *t*-test. **(B)** Immunostaining with 8-OHdG and 4-HNE in the control and pitavastatin-NP (16 mg/body of pitavastatin) groups. The bar graph indicates the percentage of 8-OHdG-positive nuclei and 4-HNE-positive areas in 5 randomly selected fields. The data are expressed as means ± SD (n = 4–5 each) and were compared using an unpaired *t*-test. Scale bar: 100 μm. **(C)** TUNEL staining of the ischemic areas in the control and pitavastatin-NP (16 mg/body of pitavastatin) groups. Nuclei are counter stained with propidium iodide (PI). The bar graph indicates the percentage of TUNEL-positive cells in 5 randomly selected fields. The data are expressed as means ± SD (n = 4–5 each) and were compared using an unpaired *t*-test. Scale bar: 200 μm. NC: negative control.

Because we previously reported that Akt phosphorylation in the IR-injured myocardium was critical for cardioprotection in a rat model[9], we examined the phosphorylation of Akt in the pig model by western blotting myocardial tissue 2 hours after reperfusion. Consistent with our previous results, pitavastatin-NP treatment induced Akt phosphorylation at Thr308 in the ischemic area (Fig 3A). Immunohistochemistry analysis of 8-OHdG and 4-HNE revealed that oxidative stress in the cardiomyocytes in the ischemic area was significantly reduced by pitavastatin-NP treatment 24 hours after reperfusion (Fig 3B). Consistently, TUNEL staining showed that apoptotic cells were significantly reduced in the ischemic area in the pitavastatin-NP-treated animals 24 hours after reperfusion (Fig 3C). These data are consistent with our previous study of pitavastatin-NP in a rat IR model[9].

### Effects of Pitavastatin-NP on Pharmacokinetics and the No-Reflow Phenomenon in Anesthetized Domestic Pigs

**Fig 4:**
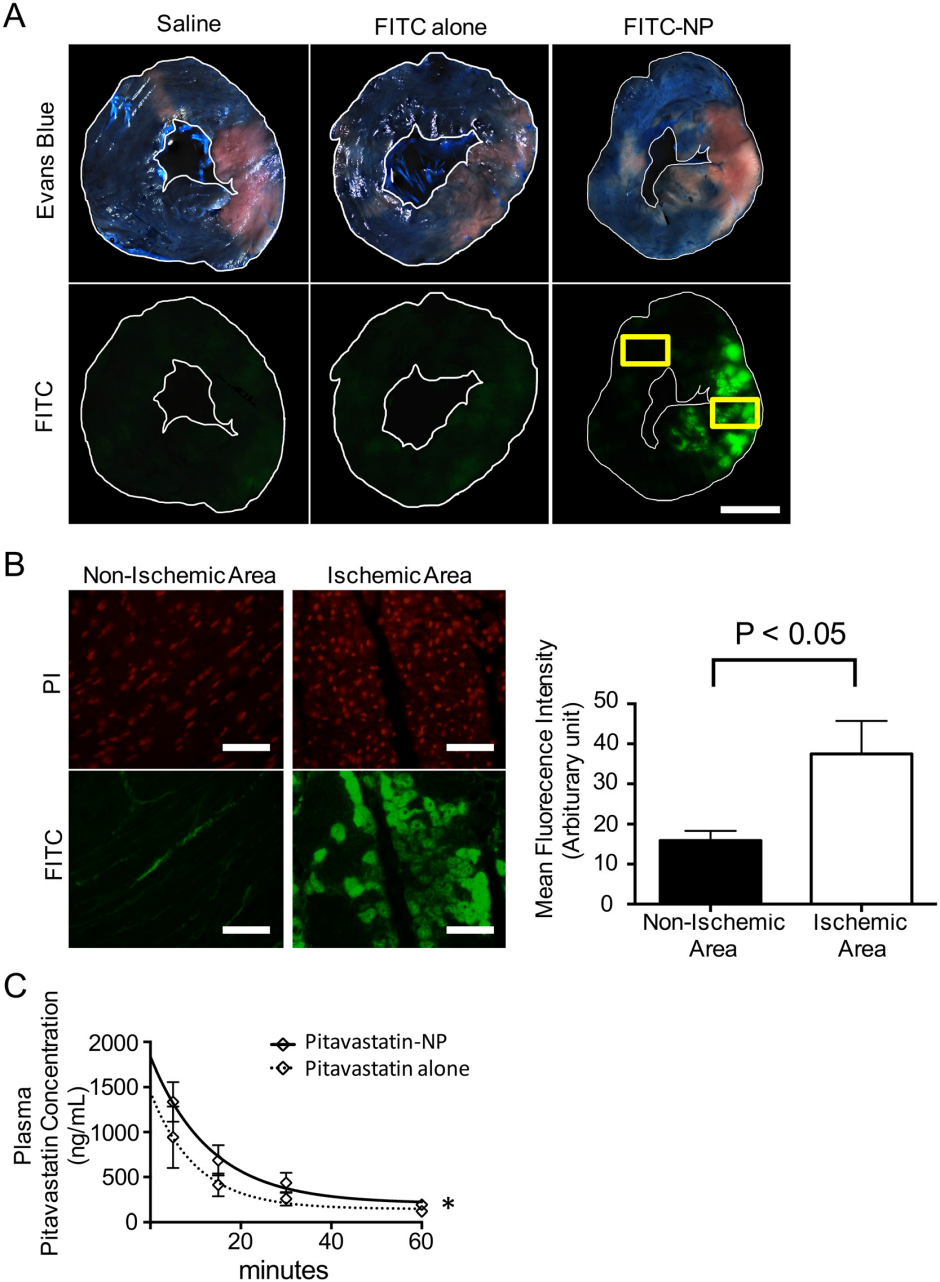
Selective Delivery of PLGA-NP to the Ischemic Myocardium and Pharmacokinetics of Pitavastatin-NP in Anesthetized Domestic Pigs. **(A)** Representative light (upper) and fluorescence (lower) images of heart cross-sections 2 hours after an intravenous injection of saline, FITC alone or FITC-NP. The Evans blue-stained areas in the upper row indicate the non-ischemic areas. FITC signals were exclusively detected in the ischemic area in the FITC-NP-treated group, whereas no FITC signal was observed in the saline- and FITC-treated groups. Scale bar: 1 cm. **(B)** Tissue sections from the ischemic and non-ischemic myocardium (yellow rectangle in (A)). FITC signals were almost exclusively detected in the ischemic myocardium. Scale bar: 50 μm. The bar graph shows the quantitation of the FITC signals of 5 randomly selected high power fields from each area. The data are expressed as means ± SD (n = 5 each) and were compared using an unpaired t-test. **(C)** Plasma concentrations of pitavastatin in pigs treated with pitavastatin-alone (16 mg/body) or pitavastatin-NP (16 mg/body of pitavastatin). Blood samples were obtained by serial sampling 5, 15, 30 and 60 minutes after reperfusion. Non-linear curve fitting was performed, and the data are expressed as means ± SD (n = 5 each). *P<0.01 between the two curves.

FITC-NP was injected 5 minutes prior to reperfusion, and the distribution of PLGA-NP in the heart was evaluated 2 hours later (S2A Fig). The FITC signal was exclusively detected in the ischemic area, whereas a faint FITC signal was observed in the non-ischemic area of the myocardium of animals treated with saline or FITC alone (Fig 4A). Histopathological examination of the heart sections detected FITC signals in the ischemic myocardium, which was more significant than in the non-ischemic myocardium (Fig 4B).

To evaluate the pharmacokinetics of intravenously injected pitavastatin-NP, serial blood sampling and biopsy of ischemic and non-ischemic myocardium was performed 5, 15, 30 and 60 minutes after injection of pitavastatin-NP or pitavastatin (S2B Fig). As we previously reported in a rat IR model[9], the plasma pitavastatin concentration immediately (5 minutes) after intravenous administration was significantly higher in the pitavastatin-NP group than in the pitavastatin-alone group, and the time-dose relationship was different (Fig 4C, Table 5). We observed no differences in the tissue concentrations of pitavastatin between the ischemic and non-ischemic myocardium in the pitavastatin-alone and pitavastatin-NP groups (Table 5).

**Table 5:** Plasma and tissue concentration of pitavastatin after intravenous administration of pitavastatin-NP or pitavastatin at the time of reperfusion.

**Pitavastatin -NP containing 16 mg pitavastatin**							
Groups	Time after intravenous administration				T_1/2_ (min)	Cmax	AUC
	5 min	15 min	30 min	60 min			
Non-ischemic Area	672 ± 114	478 ± 139	249 ±66	155 ± 84	95.5 ± 163	683 ± 94	315 ± 62
Ischemic Area	559 ± 182	415 ± 36	381 ± 75	305 ± 29	116 ± 46.6	563 ± 176	375 ± 32
Plasma	1335 ± 221*	686 ± 169	435 ± 113	193 ± 32	25.6 ± 4.02	941 ± 341	521 ± 106*
**16 mg pitavastatin**							
Groups	Time after intravenous administration				T_1/2_ (min)	Cmax	AUC
	5 min	15 min	30 min	60 min			
Non-ischemic Area	415 ± 87	250± 84	145 ± 51	190 ± 144	98.5 ± 117	415 ± 86	205 ± 73
Ischemic Area	434 ± 203	431 ± 76	325 ± 166	264 ± 145	76.1 ± 45.5	558 ± 90	331 ± 91
Plasma	941 ± 342	412 ± 127	258 ± 75	119 ± 55	25.5 ± 5.05	1335 ± 221	329 ± 93

Data are expressed as mean ± SD (ng/g・tissue for myocardial tissue and ng/mL for plasma, ng・hr/g・tissue for AUC of myocardial tissue, ng・hr/mL for AUC of plasma, n = 5 each).

* P<0.01 versus pitavastatin group by 2way ANOVA followed by Sidak’s multiple comparisons test.

**Fig 5:**
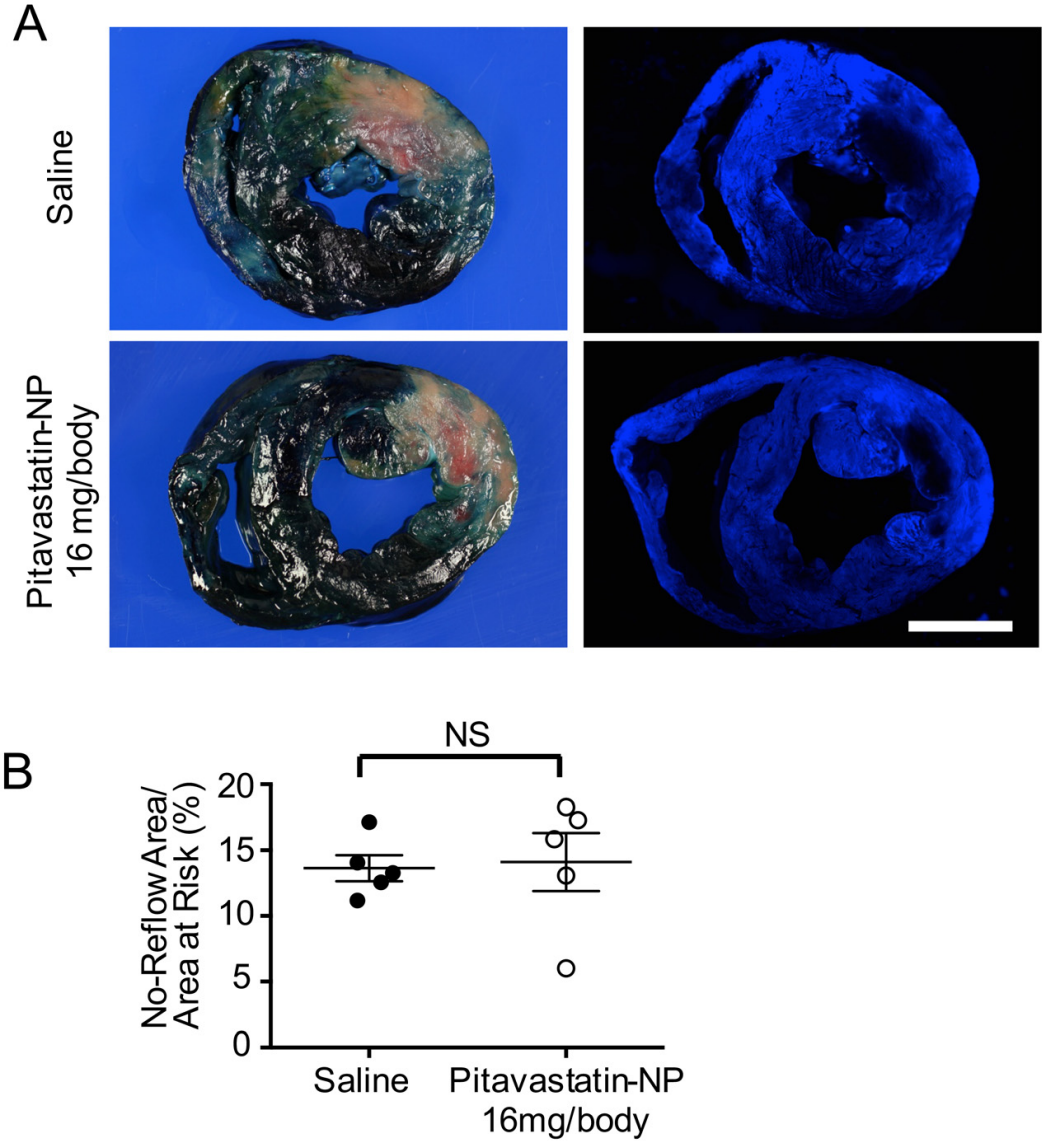
The Effect of Pitavastatin-NP on the No-Reflow Phenomenon in Anesthetized Domestic Pigs. **(A)** Representative light (left) and fluorescence (right) images of heart cross-sections 2 hours after intravenous injections of saline or pitavastatin-NP (16 mg/body of pitavastatin). The Evans blue-stained areas stained in the left row indicate the non-ischemic areas, and the pale areas indicate the ischemic areas. The black area in the right row indicates the no-reflow area. Scale bar: 1 cm. **(B)** The effects of pitavastatin-NP on the no-reflow area. The data are expressed as means ± SEM (n = 5 each) and were compared using an unpaired *t*-test.

The area of no-reflow was quantified by thioflavin-S staining 2 hours after reperfusion (S2C Fig). Treatment with pitavastatin-NP containing 16 mg/body pitavastatin did not affect the areas of no-reflow or areas at risk compared with the saline-treated group (Fig 5).

### Effects of Pitavastatin-NP on Cardiomyocyte Cell Death

**Fig 6:**
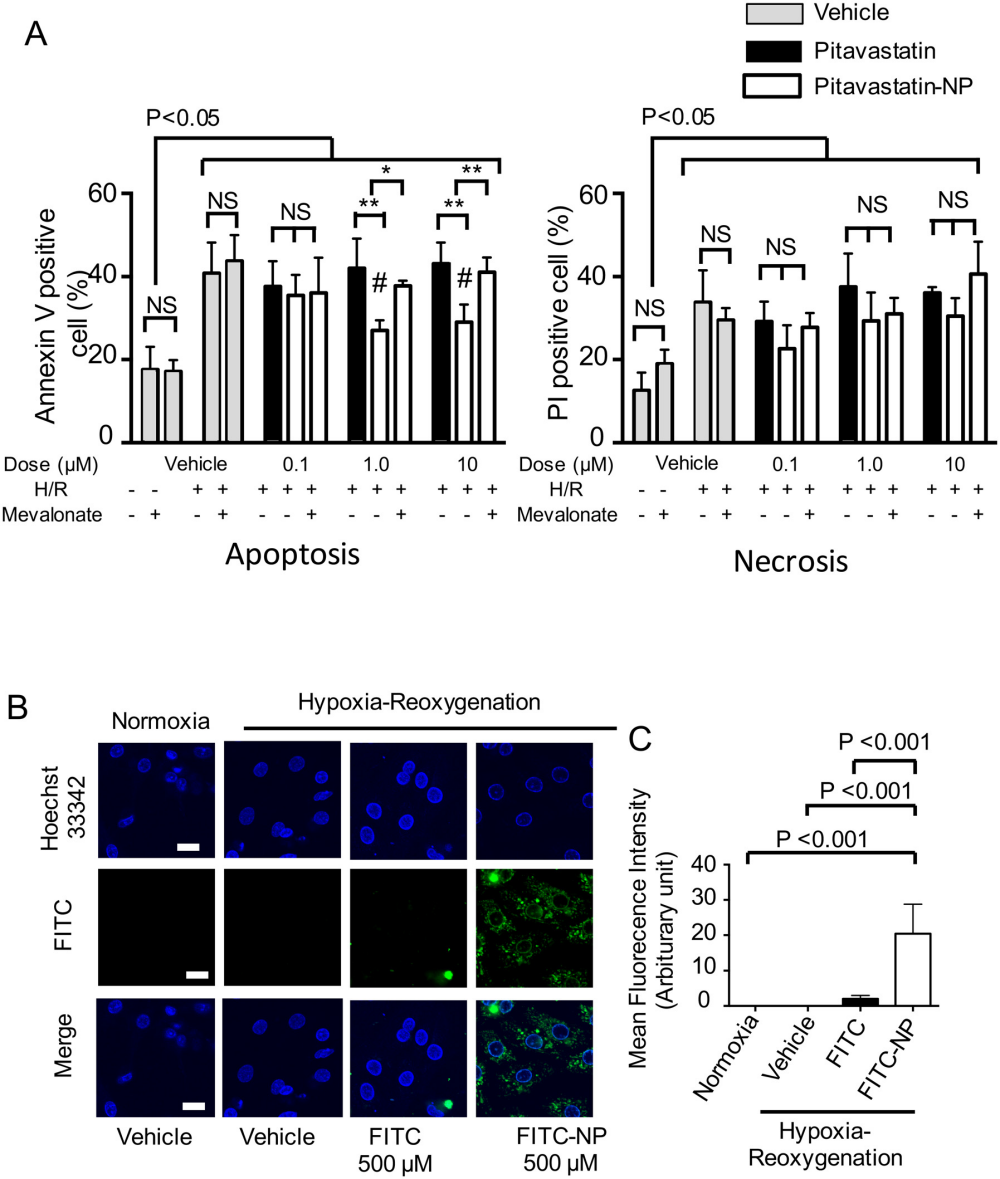
The Effect of Pitavastatin-NP on Cultured Cardiomyocytes Subjected to Hypoxia/Reoxygenation. **(A)** Cultured cardiomyocytes were subjected to hypoxia/reoxygenation (H/R), and cell death was characterized as apoptosis (annexin V FITC-positive/PI-negative) or necrosis (PI-positive), as determined by flow cytometry. The white bars indicate the pitavastatin-NP-treated groups. The data are expressed as means ± SD (N = 6 each). *P<0.05, **P<0.01 between the two groups indicated by the bar, as determined by two-way ANOVA followed by Sidak’s multiple comparison test. #P<0.01 versus the vehicle group with H/R, as determined by two-way ANOVA followed by Sidak’s multiple comparison test. **(B)** Confocal microscopy of neonatal rat ventricular cardiomyocytes subjected to H/R. The cells were co-incubated with vehicle, FITC or FITC-NP, and the nuclei were stained with Hoechst 33342 (blue). Scale bar: 20 μm. **(C)** Quantitation of the FITC signals of 5 randomly selected microscopic fields of view. The data are expressed as means ± SD and were compared using one-way ANOVA followed by Dunnett’s multiple comparison test.

Cell death was induced by hypoxia in cultured cardiomyocytes, followed by reoxygenation. The cells were treated with vehicle, pitavastatin (0.1, 1.0 or 10 μmol/L) or pitavastatin-NP (0.1, 1.0 or 10 μmol/L of pitavastatin) at the time of reoxygenation. Cell death was characterized as apoptosis (annexin V-FITC staining) or necrosis (PI staining) by flow cytometry. Pitavastatin-NP at doses of 1.0 μmol/L and 10 μmol/L significantly reduced the rate of apoptosis of cardiomyocytes undergoing hypoxia/reoxygenation, but pitavastatin alone at the same dose or pitavastatin-NP at a dose of 0.1 μmol/L did not produce the same effect (Fig 6A). This anti-apoptotic effect was inhibited by co-treatment with mevalonic acid (100 μmol/L). Although there was a trend that pitavastatin-NP reduced the rate of necrosis compared with pitavastatin alone at each dose, this difference did not reach statistical significance (Fig 6A).

To determine the intracellular localization of the nanoparticles, cardiomyocytes were treated with FITC or FITC-NP. Cardiomyocytes treated with FITC-NP had a significantly stronger intracellular FITC fluorescence intensity compared with those treated with the same amount of FITC alone (Fig 6B and 6C).

## Discussion

In the present study, we performed a preclinical proof-of-concept study in a conscious mini-pig myocardial IR model and found that treatment with pitavastatin-NP protects the heart from IR injury without apparent adverse side effects. In addition, pitavastatin-NP tended to attenuate post-infarction left ventricular remodeling and improved cardiac function.

The mini-pig myocardial IR injury model presented here showed stable hemodynamics, without abnormal changes in blood pressure and heart rate, as well as a low incidence of fatal arrhythmia and low mortality during the experimental period. Our data suggest that the efficacy and safety of pitavastatin-NP in our conscious mini-pig model are highly reliable, and this new model is a more appropriate and feasible preclinical model for IR injury compared with anesthetized large animal models. In the present study, we found that pitavastatin-NP reduced the MI size at doses of 8, 16 and 32 mg/body, suggesting that an appropriate dose of pitavastatin-NP in human patients would range from 8 to 16 mg/body. In addition, cardiac MRI performed 4 weeks after IR injury demonstrated that treatment with pitavastatin-NP decreased the left ventricular end systolic volume and increased left ventricular ejection fraction compared with those on treatment with saline. However, the effects of pitavastatin-NP on left ventricular end diastolic volume did not achieve statistical significance. It is known that significant post-MI left ventricular remodeling (left ventricular dilatation associated with increased end diastolic volume) develops in animals and humans when more than 30% of the left ventricle fell into MI[30,31]. The observed MI size in the none-treated control group was small (19 ± 7% of LV), which might lead to inadequate left ventricular remodeling. Therefore, further studies are needed to examine the effects of pitavastatin-NP on left ventricular remodeling and function during the chronic phases of IR injury.

Our present pharmacokinetic data in an anesthetized porcine model, showing higher plasma concentration of pitavastatin immediately after intravenous administration in the pitavastatin-NP group than in the pitavastatin-alone group, are comparable to our previous reports in a rat IR model[9]. These data are also supported by prior reports demonstrating that PLGA nanoparticles prolong the in vivo circulation time of the nanoparticulated drug from minutes to several hours after intravenous administration [9,10,32]. However, the observed tissue concentrations of pitavastatin between the ischemic and non-ischemic myocardium does not account for the superior therapeutic effect of pitavastatin-NP. Although the therapeutic target of statins is known to be an intracellular 3-hydroxy-3-methylglutaryl coenzyme-A reductase, it is impossible to measure intracellular concentration of pitavastatin or its metabolite in cardiomyocytes or inflammatory cells *in vivo* by state-of-art methods. Therefore, further studies are needed to examine whether intracellular concentrations of pitavastatin are greater in cardiomyocytes and inflammatory cells in the ischemic myocardium than in those in the non-ischemic myocardium. In addition, thioflavin-S staining suggested that pitavastatin-NP showed no effect on the no-reflow phenomenon.

We have previously reported that PLGA nanoparticles are selectively delivered to cardiomyocytes and inflammatory cells (mainly monocytes) in IR-injured myocardium after an intravenous injection at the time of reperfusion[8,9]. Moreover, our results suggested that PLGA nanoparticles are a clinically feasible drug delivery system for IR injury. We also reported that the cardioprotective effect of pitavastatin-NP on IR injury was mediated by inhibiting monocyte-mediated inflammation as well as the resulting cardiomyocyte apoptosis occurring during the late phase of reperfusion in a rat model[9]. The cardioprotective effects of pitavastatin-NP on IR injury in wild-type mice also appear to be blunted in CCR2-deficient mice (unpublished observations by our laboratory). Overall, these data in small animal models suggest that monocyte-mediated inflammation plays a central role in the mechanism by which pitavastatin-NP exerts cardioprotection against IR injury induced by MI. Although we could not show direct evidence of reduced inflammation in the IR myocardium in the present study due to a lack of antibodies that cross react with porcine monocytes, we showed that pitavastatin-NP reduced oxidative stress, which is associated with inflammation[33], in the IR-injured myocardium. Inflammatory cells recruited into IR-injured myocardium generate reactive oxygen species and stimulate the release of pro-apoptotic factors in the ischemic myocardium[34], resulting in cardiomyocyte apoptosis after IR[33]. In the present study, we showed that PLGA nanoparticles are incorporated by cultured cardiomyocytes under hypoxia-reoxygenation, and pitavastatin-NP exerts a direct anti-apoptotic effect on cultured cardiomyocytes undergoing hypoxia-reoxygenation. However, further studies are needed to determine the mechanism by which pitavastatin-NP exerts its anti-apoptotic effect on IR injury in vivo.

In summary, treatment with pitavastatin-NP exerts cardioprotective effects on IR injury without apparent adverse side effects in a preclinical conscious porcine model. This nanotechnology-based approach can be developed as a novel therapeutic strategy for the treatment of patients presenting with AMI. Indeed, we have conducted phase I clinical trials (UMIN000014940, UMIN000019189) of intravenous injections of pitavastatin-NP. We are also conducting a phase I/IIa clinical trial (UMIN000008011) of intramuscular injections of pitavastatin-NP in patients with critical limb ischemia. We are now planning to perform a phase IIa clinical trial for patients with AMI.

## Supporting Information

S1 Fig Experimental Protocols. **(A)** Experimental protocol 1: Protocol for measuring the MI size in mini-pigs. Five minutes prior to reperfusion, the pigs were divided into four groups treated intravenously with the following drugs: 1) saline (10 mL/body), 2) FITC-NP (PLGA containing 10 mg of FITC in saline; 10 mL/body), 3) pitavastatin alone (8 mg in saline; 10 mL/body), or 4) pitavastatin-NP (PLGA containing 4, 8, 16, or 32 mg of pitavastatin in saline; 10 mL/body). Before and during the ischemia-reperfusion procedure, an electrocardiogram at the left precordial lead, arterial blood pressure, heart rate and body temperature were continuously monitored using a telemetry system without administering anesthesia. Blood sampling was performed at baseline (1 hour prior to ischemia) and 24 hours after reperfusion. To measure the MI size, the animals were euthanized 24 hours after reperfusion with an overdose of pentobarbital, and the hearts were excised. The LCx was re-occluded, and the MI size was measured using 1.0% Evans blue and 1.0% triphenyltetrazolium chloride staining. **(B)** Experimental protocol 2: Western blot protocol for the samples from the mini-pigs. Four pigs treated with FITC-NP (PLGA containing 10 mg of FITC in saline; 10 mL/body) and 5 pigs treated with pitavastatin-NP (PLGA containing 16 mg of pitavastatin in saline; 10 mL/body) were euthanized 2 hours after reperfusion, and tissues were harvested from the non-ischemic and ischemic areas. **(C)** Experimental protocol 3: Immunohistochemistry and TUNEL staining protocols for the samples from the mini-pigs. Four pigs treated with FITC-NP (PLGA containing 10 mg of FITC in saline; 10 mL/body) and 5 pigs treated with pitavastatin-NP (PLGA containing 16 mg of pitavastatin in saline; 10 mL/body) were euthanized 24 hours after reperfusion, and tissues were harvested from the non-ischemic and ischemic areas. **(D)** Experimental protocol 4: Protocol for cardiac MRI in mini-pigs. Eight pigs each from the saline- (10 mL/body) and pitavastatin-NP-treated (PLGA containing 16 mg of pitavastatin in saline; 10 mL/body) groups were monitored until 4 weeks after reperfusion, and cardiac MRI was performed to assess cardiac function and left ventricular remodeling.
(TIF)

S2 Fig Experimental Protocols. **(A)** Experimental protocol 5: Protocol for the domestic pigs used for the tracing study. Two pigs each were treated with either saline, FITC (0.33 mg/kg in saline; 10 mL/body), or FITC-NP (PLGA containing 0.33 mg/kg FITC in saline; 10 mL/body) 5 minutes before reperfusion, and 2 hours after reperfusion, the pigs were euthanized, and the hearts were excised. The LCx was re-occluded, and 1.0% Evans blue was injected into both coronary arteries. The left ventricles were sectioned into 10-mm-thick cross-sectional myocardial slices and were photographed by fluorescence stereomicroscopy. **(B)** Experimental protocol 6: Protocol for domestic pigs used for the pharmacokinetic study. Pitavastatin (16 mg/body) or pitavastatin-NP (16 mg/body) were intravenously injected 5 minutes prior to reperfusion. Serial blood sampling and biopsy of the ischemic and non-ischemic myocardium were performed 5, 15, 30 or 60 minutes after reperfusion to measure the plasma and tissue concentrations of pitavastatin. **(C)** Experimental protocol 7: Protocol for domestic pigs used to assess the no-reflow areas. Pigs treated with saline (10 mL/body) or pitavastatin-NP (PLGA containing 16 mg of pitavastatin in saline; 10 mL/body) were reperfused for 2 hours, and 4% thioflavin S was injected into the left coronary artery via a 7-Fr catheter. Then, the animals were euthanized, and the heart was excised. The LCx was re-occluded, and 1% Evans blue was injected into both coronary arteries. The left ventricles were sectioned into 10-mm-thick cross-sectional myocardial slices and were photographed with a digital camera.
(TIF)

S3 Fig Scheme illustrating the method for grouping the experimental animals. **(A)** Mini-pigs used to measure the MI size. **(B)** Mini-pigs used for western blot, immunohistochemistry and TUNEL staining. **(C)** Mini-pigs used for cardiac MRI. **(D)** Domestic pigs used for the tracing and pharmacokinetic studies. **(E)** Domestic pigs used to assess the no-reflow areas.
(TIF)

S4 Fig Effects of Saline, FITC-NP, Pitavastatin and Pitavastatin-NP on MI Size in Mini-Pigs. **(A)** The effects of saline (n = 7) and FITC-NP (n = 4) on MI size. The data are expressed as means ± SEM and were compared using an unpaired *t*-test. **(B)** The area at risk as a percentage of the left ventricle (LV). The data are expressed as means ± SEM (n = 4–7 each) and were compared using an unpaired *t*-test. **(C)** The effects of saline (n = 7), pitavastatin (8 mg/body, n = 3) and pitavastatin-NP (8 mg/body of pitavastatin, n = 7) on MI size. The data are expressed as means ± SEM and were compared using one-way ANOVA followed by Tukey’s multiple comparison test. **(D)** The area at risk as a percentage of the left ventricle (LV). The data are expressed as means ± SEM (n = 3–7 each) and were compared using one-way ANOVA followed by Tukey’s multiple comparison test.
(TIF)

S5 Fig Flow Cytometric Analysis of Cell Death in Cultured Cardiomyocytes. **(A)** Gating strategy to identify the apoptotic and necrotic cells in cultured cardiomyocytes. Apoptosis was defined as annexin V FITC-positive/propidium iodide (PI)-negative cells, whereas necrosis was defined as PI-positive cells. **(B)** Representative dot plots and histograms of cells treated with vehicle, pitavastatin alone (10 μmol/L) or pitavastatin-NP (10 μmol/L) with or without mevalonic acid (100 μmol/L).
(TIF)
