# Supplementary figures and images for: A Translational Study of a New Therapeutic Approach for Acute Myocardial Infarction: Nanoparticle-Mediated Delivery of Pitavastatin into Reperfused Myocardium Reduces Ischemia-Reperfusion Injury in a Preclinical Porcine Model
Source: PLoS One. 2016 Sep 7;11(9):e0162425. doi: 10.1371/journal.pone.0162425 (PMC5014419; doi:10.1371/journal.pone.0162425)

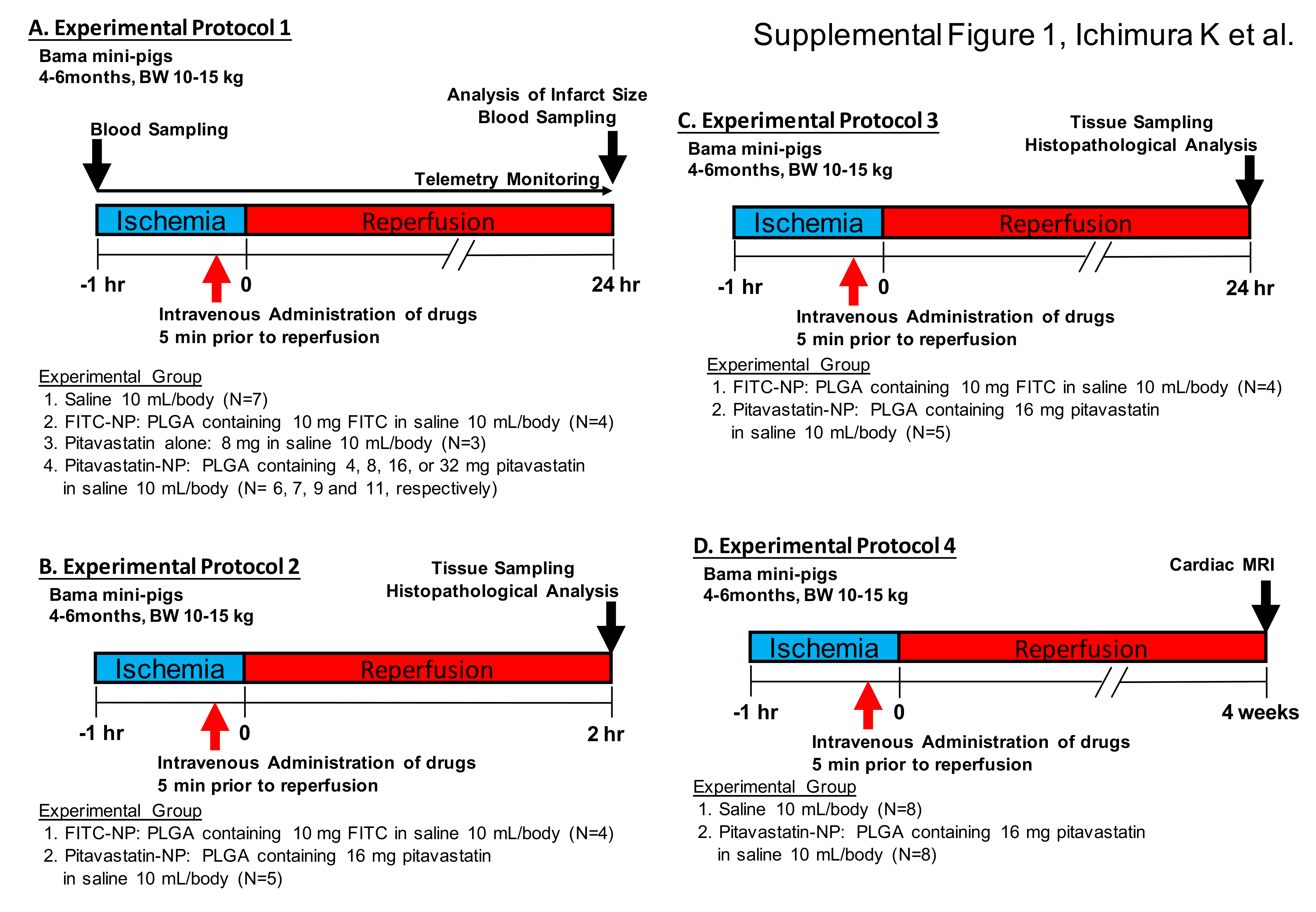

Supplement: S1 Fig — (A) Experimental protocol 1: Protocol for measuring the MI size in mini-pigs. Five minutes prior to reperfusion, the pigs were divided into four groups treated intravenously with the following drugs: 1) saline (10 mL/body), 2) FITC-NP (PLGA containing 10 mg of FITC in saline; 10 mL/body), 3) pitavastatin alone (8 mg in saline; 10 mL/body), or 4) pitavastatin-NP (PLGA containing 4, 8, 16, or 32 mg of pitavastatin in saline; 10 mL/body). Before and during the ischemia-reperfusion procedure, an electrocardiogram at the left precordial lead, arterial blood pressure, heart rate and body temperature were continuously monitored using a telemetry system without administering anesthesia. Blood sampling was performed at baseline (1 hour prior to ischemia) and 24 hours after reperfusion. To measure the MI size, the animals were euthanized 24 hours after reperfusion with an overdose of pentobarbital, and the hearts were excised. The LCx was re-occluded, and the MI size was measured using 1.0% Evans blue and 1.0% triphenyltetrazolium chloride staining. (B) Experimental protocol 2: Western blot protocol for the samples from the mini-pigs. Four pigs treated with FITC-NP (PLGA containing 10 mg of FITC in saline; 10 mL/body) and 5 pigs treated with pitavastatin-NP (PLGA containing 16 mg of pitavastatin in saline; 10 mL/body) were euthanized 2 hours after reperfusion, and tissues were harvested from the non-ischemic and ischemic areas. (C) Experimental protocol 3: Immunohistochemistry and TUNEL staining protocols for the samples from the mini-pigs. Four pigs treated with FITC-NP (PLGA containing 10 mg of FITC in saline; 10 mL/body) and 5 pigs treated with pitavastatin-NP (PLGA containing 16 mg of pitavastatin in saline; 10 mL/body) were euthanized 24 hours after reperfusion, and tissues were harvested from the non-ischemic and ischemic areas. (D) Experimental protocol 4: Protocol for cardiac MRI in mini-pigs. Eight pigs each from the saline- (10 mL/body) and pitavastatin-NP-treated ( [file pone.0162425.s001.tif]

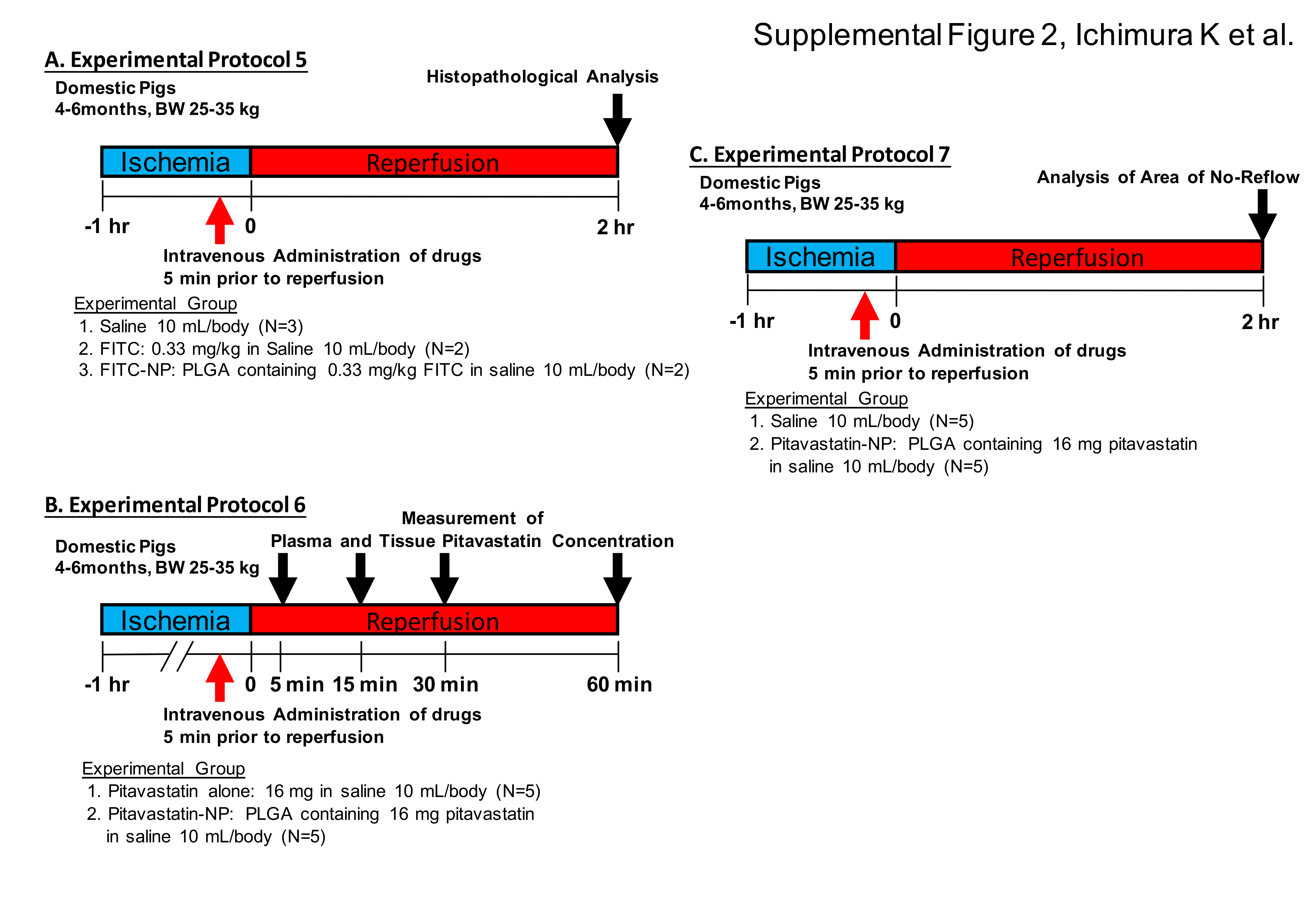

Supplement: S2 Fig — (A) Experimental protocol 5: Protocol for the domestic pigs used for the tracing study. Two pigs each were treated with either saline, FITC (0.33 mg/kg in saline; 10 mL/body), or FITC-NP (PLGA containing 0.33 mg/kg FITC in saline; 10 mL/body) 5 minutes before reperfusion, and 2 hours after reperfusion, the pigs were euthanized, and the hearts were excised. The LCx was re-occluded, and 1.0% Evans blue was injected into both coronary arteries. The left ventricles were sectioned into 10-mm-thick cross-sectional myocardial slices and were photographed by fluorescence stereomicroscopy. (B) Experimental protocol 6: Protocol for domestic pigs used for the pharmacokinetic study. Pitavastatin (16 mg/body) or pitavastatin-NP (16 mg/body) were intravenously injected 5 minutes prior to reperfusion. Serial blood sampling and biopsy of the ischemic and non-ischemic myocardium were performed 5, 15, 30 or 60 minutes after reperfusion to measure the plasma and tissue concentrations of pitavastatin. (C) Experimental protocol 7: Protocol for domestic pigs used to assess the no-reflow areas. Pigs treated with saline (10 mL/body) or pitavastatin-NP (PLGA containing 16 mg of pitavastatin in saline; 10 mL/body) were reperfused for 2 hours, and 4% thioflavin S was injected into the left coronary artery via a 7-Fr catheter. Then, the animals were euthanized, and the heart was excised. The LCx was re-occluded, and 1% Evans blue was injected into both coronary arteries. The left ventricles were sectioned into 10-mm-thick cross-sectional myocardial slices and were photographed with a digital camera. (TIF) [file pone.0162425.s002.tif]

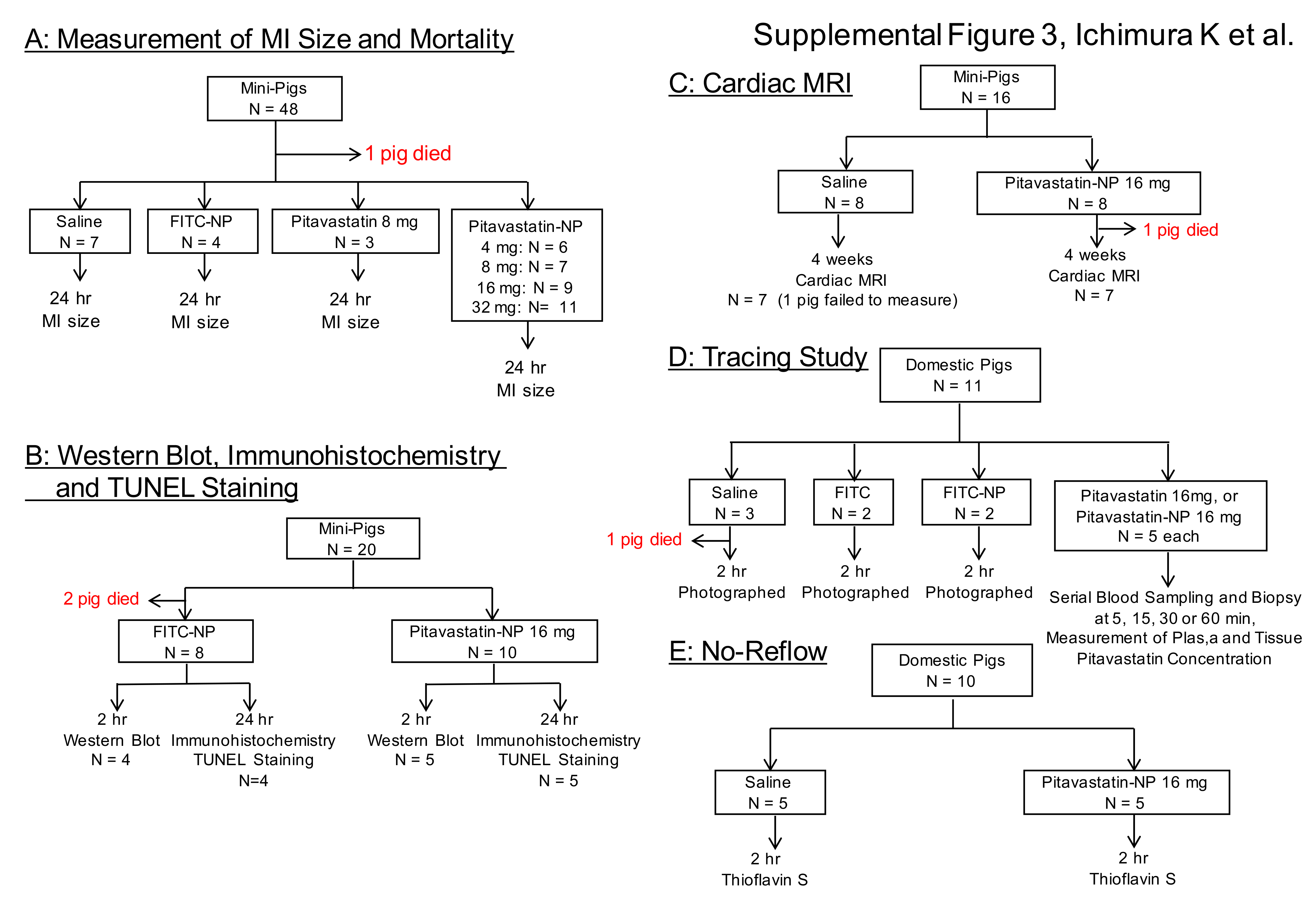

Supplement: S3 Fig — (A) Mini-pigs used to measure the MI size. (B) Mini-pigs used for western blot, immunohistochemistry and TUNEL staining. (C) Mini-pigs used for cardiac MRI. (D) Domestic pigs used for the tracing and pharmacokinetic studies. (E) Domestic pigs used to assess the no-reflow areas. (TIF) [file pone.0162425.s003.tif]

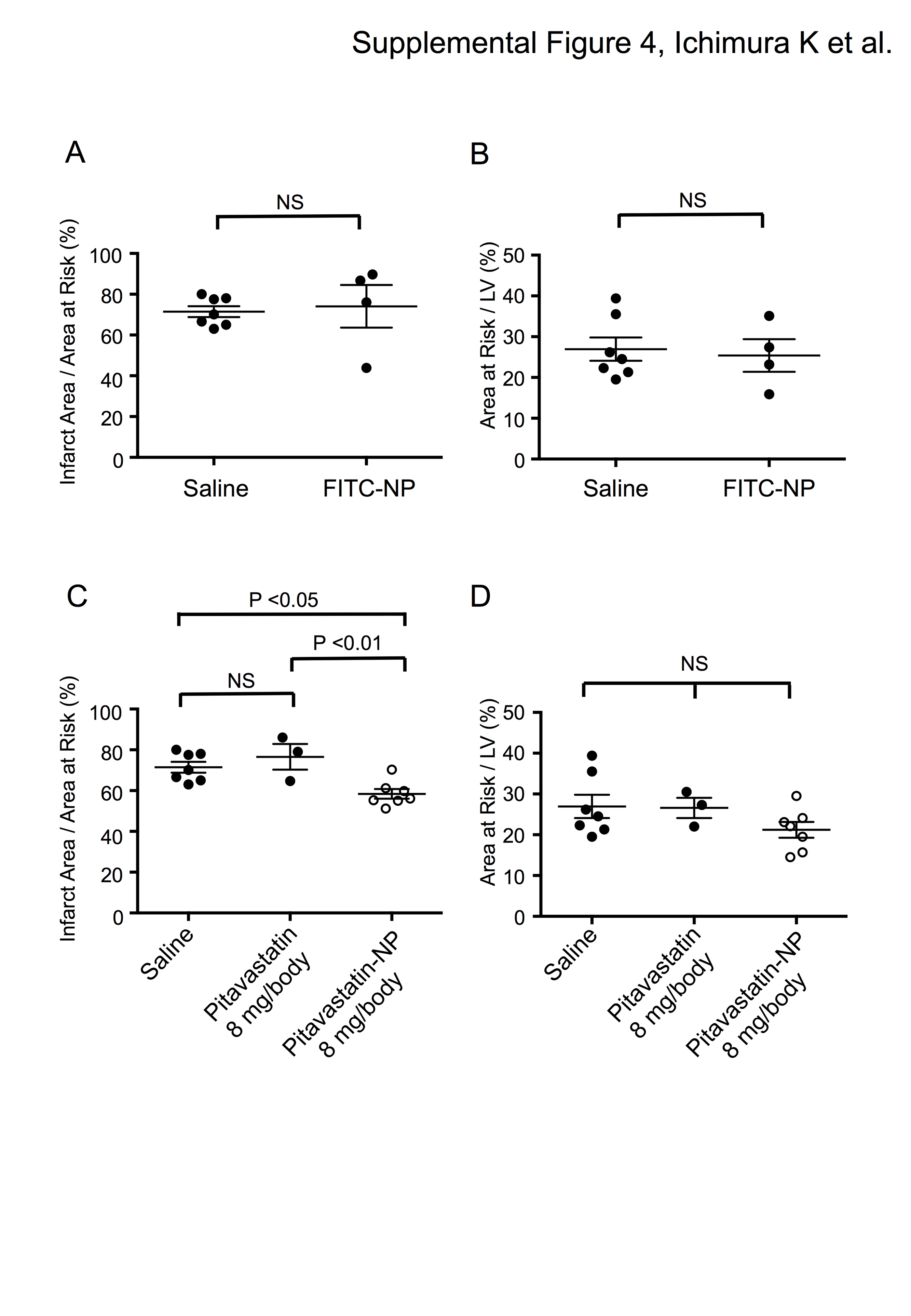

Supplement: S4 Fig — (A) The effects of saline (n = 7) and FITC-NP (n = 4) on MI size. The data are expressed as means ± SEM and were compared using an unpaired t-test. (B) The area at risk as a percentage of the left ventricle (LV). The data are expressed as means ± SEM (n = 4–7 each) and were compared using an unpaired t-test. (C) The effects of saline (n = 7), pitavastatin (8 mg/body, n = 3) and pitavastatin-NP (8 mg/body of pitavastatin, n = 7) on MI size. The data are expressed as means ± SEM and were compared using one-way ANOVA followed by Tukey’s multiple comparison test. (D) The area at risk as a percentage of the left ventricle (LV). The data are expressed as means ± SEM (n = 3–7 each) and were compared using one-way ANOVA followed by Tukey’s multiple comparison test. (TIF) [file pone.0162425.s004.tif]

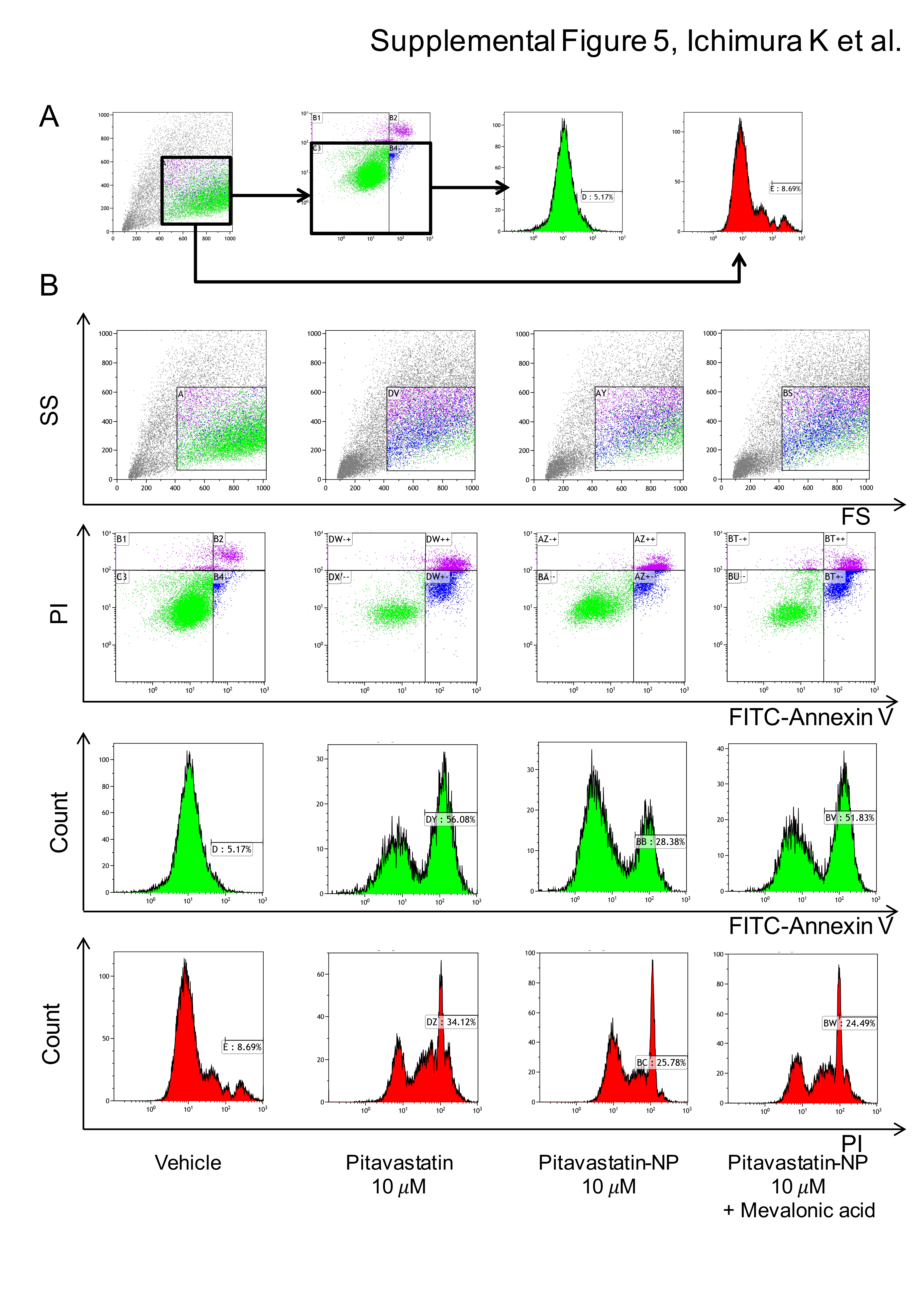

Supplement: S5 Fig — (A) Gating strategy to identify the apoptotic and necrotic cells in cultured cardiomyocytes. Apoptosis was defined as annexin V FITC-positive/propidium iodide (PI)-negative cells, whereas necrosis was defined as PI-positive cells. (B) Representative dot plots and histograms of cells treated with vehicle, pitavastatin alone (10 μmol/L) or pitavastatin-NP (10 μmol/L) with or without mevalonic acid (100 μmol/L). (TIF) [file pone.0162425.s005.tif]
